# Correction: RNAi-Dependent and Independent Control of LINE1 Accumulation and Mobility in Mouse Embryonic Stem Cells

**DOI:** 10.1371/journal.pgen.1005247

**Published:** 2015-05-14

**Authors:** Constance Ciaudo, Florence Jay, Ikuhiro Okamoto, Chong-Jian Chen, Alexis Sarazin, Nicolas Servant, Emmanuel Barillot, Edith Heard, Olivier Voinnet

Panel A in Fig 4 and panels A and F in S4 Fig are not presented correctly. The lanes in the corrected figures are separated by tracks for other mutants that are not relevant for the current work and can be seen in the original blots, provided here as S7 and S8 Figs. The authors apologise for the mistake and have provided corrected versions, along with the original blots that were used to create the figures. These errors do not affect the conclusions of this article.

**Fig 4:**
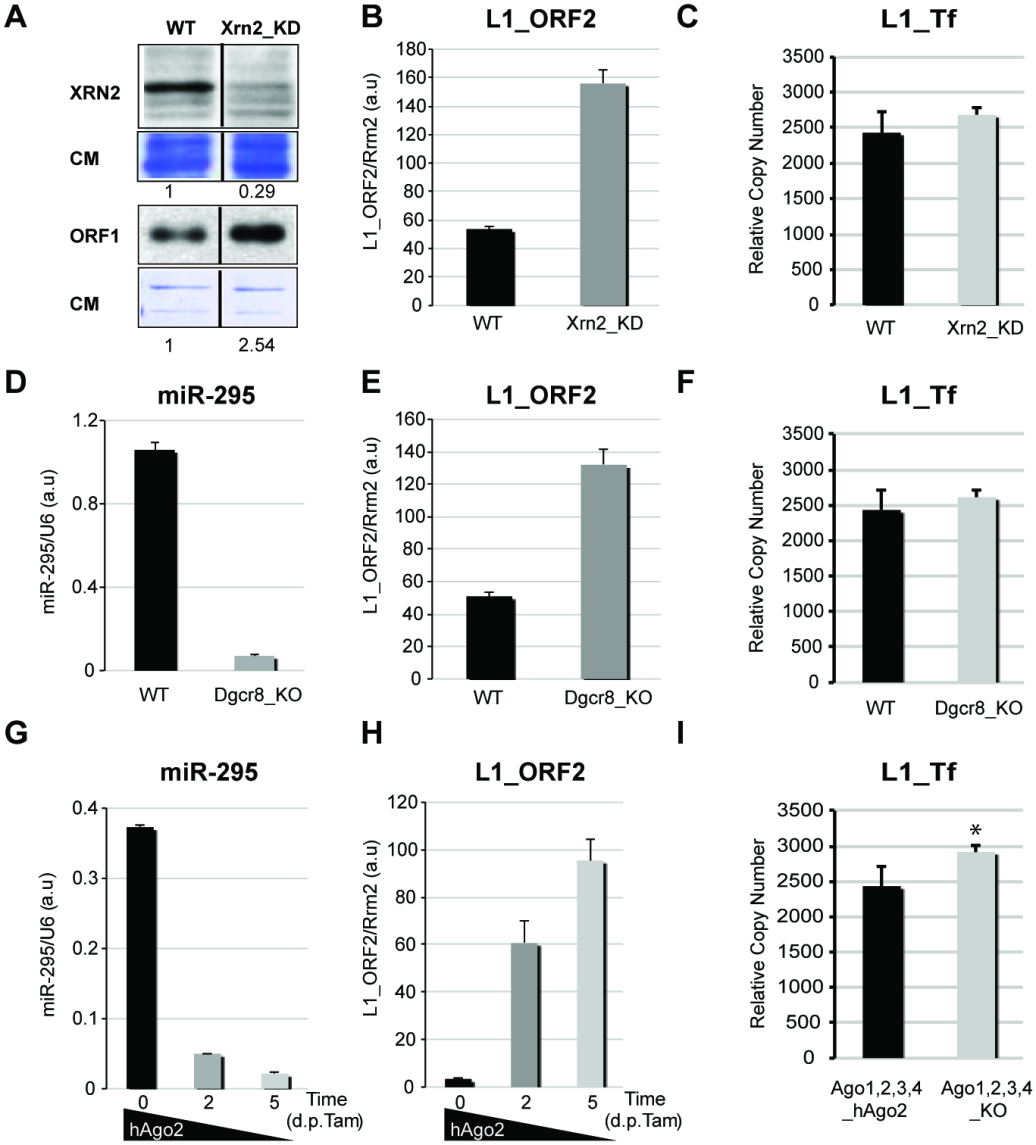
L1 mRNA levels and genomic copy-number in various knock-out and knock-down mESC lines. A. Western analysis of XRN2 and L1_ORF1 accumulation in WT and *Xrn2*_KD mESCs; CM: Coomassie staining of total protein. B. qRT-PCR analysis of L1_ORF2 mRNA levels in WT and *Xrn2*_KD mESCs. C. qPCR analysis of L1_Tf copy-number in WT and *Xrn2*_KD mESCs. D–E. qRT-PCR analysis of miR-295 (D) and L1_ORF2 mRNA (E) levels in WT and *Dgcr8*_KO mESCs. F. qPCR analysis of L1_Tf copy-number in WT and *Dgcr8*_KO mESCs. G–H. qRT-PCR analysis of miR-295 (G) and L1_ORF2 mRNA (H) levels upon hAgo2 deletion in Tamoxifen-treated *Ago1*,*2*,*3*,*4_KO* mESCs. I. qPCR analysis of L1_Tf copy-number in *Ago1*,*2*,*3*,*4_KO_hAgo2* mESCs before and after hAgo2 deletion. *: p-value<0.1.

## Supporting Information

S4 Fig L1 expression and genomic copy-number in various knock-out and knock-down mESC lines. A. Western analysis of RRP6 and L1_ORF1 accumulation in WT and *Rrp6*_KD mESCs; CM: Coomassie staining of total protein. B. Accumulation of Tf_5′-UTR (+) and (−) sRNAs detected by qRT-PCR in WT and *Xrn2*_KD mESCs. C. qPCR analysis of L1_Tf copy-number in WT and *Rrp6*_KD mESCs. D. L1_ORF2, Tf, Gf and A sub-type mRNAs accumulation detected by qRT-PCR in *Xrn2*_KD and *Rrp6*_KD mESCs. E. Accumulation of miR-320 detected by qRT-PCR in WT and *Dgcr8*_KO mESCs. F. Western analysis of AGO2 accumulation in WT and *Ago1*,*2*,*3*,*4_KO_hAgo2* mESCs before and after hAgo2 deletion induced by tamoxifen; CM: Coomassie staining of total protein. G. Accumulation of the Hmga2 and Btg2 mRNAs, respectively targeted by mmu-miR-196a and mmu-let-7a/mmu-miR-132, analyzed by qRT-PCR before and after deletion of h*Ago2*. H. mRNA accumulation of L1_Tf, _Gf and _A sub-types detected by qRT-PCR before and after h*Ago2* deletion. I. mRNA accumulation of a single Tf_L1 subtype located on chromosome 17, analyzed by semi-quantitative RT-PCR before and after h*Ago2* deletion.
(TIF)

S7 Fig Original Blots for Fig 4A. The red rectangles highlight the part of the gel presented in Fig 4A. CM: Coomassie staining of total protein.
(TIF)

S8 Fig Original Blots for S4A and S4F Fig. A. Original blot for the S4A Fig. The red rectangles highlight the part of the gel presented in S4A Fig. B. The original blot for the S4F Fig is presented. CM: Coomassie staining of total protein.
(TIF)
