# Supplementary figures and images for: Correction: RNAi-Dependent and Independent Control of LINE1 Accumulation and Mobility in Mouse Embryonic Stem Cells
Source: PLoS Genet. 2015 May 14;11(5):e1005247. doi: 10.1371/journal.pgen.1005247 (PMC4431862; doi:10.1371/journal.pgen.1005247)

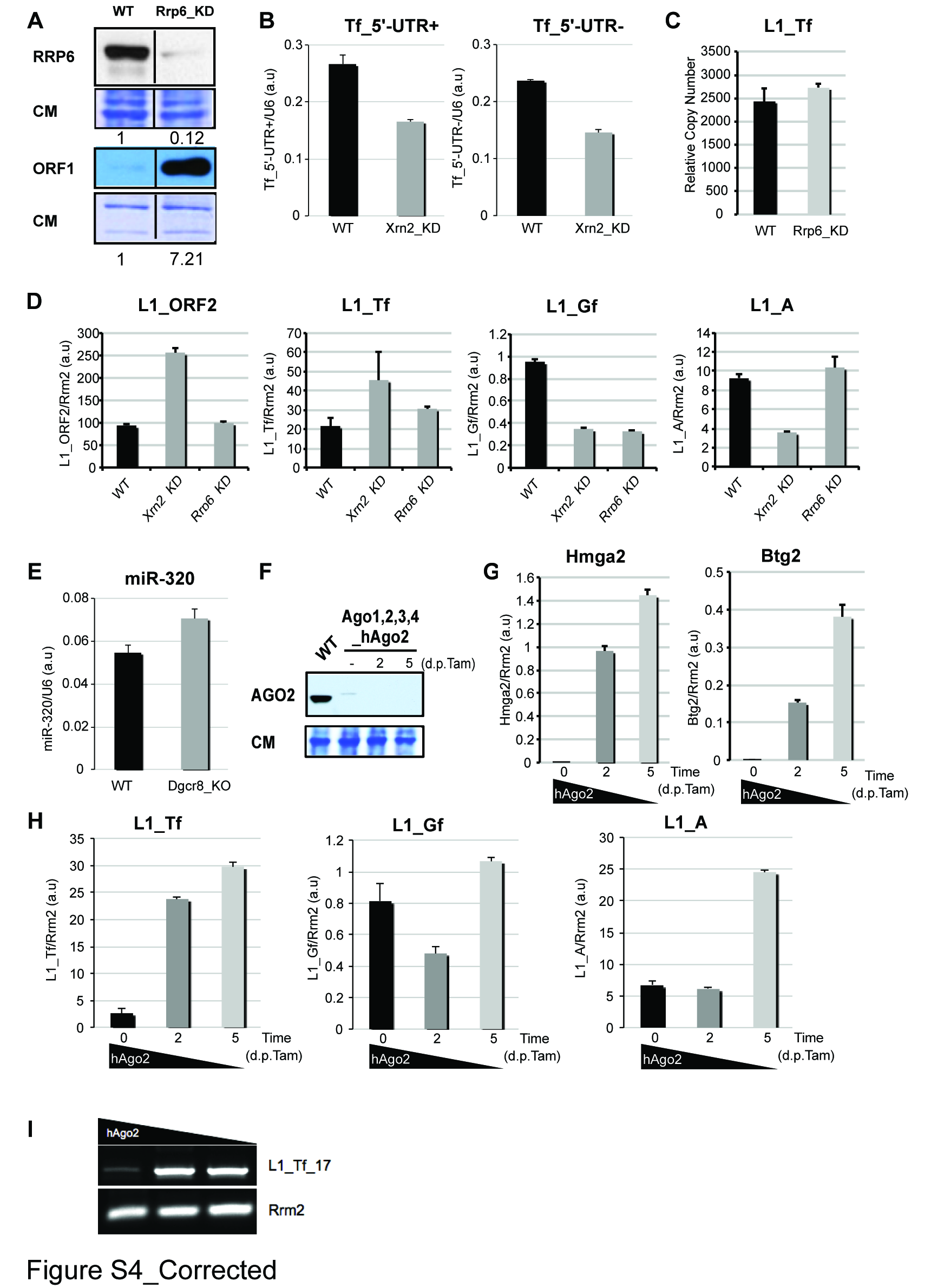

Supplement: S4 Fig — A. Western analysis of RRP6 and L1_ORF1 accumulation in WT and Rrp6_KD mESCs; CM: Coomassie staining of total protein. B. Accumulation of Tf_5′-UTR (+) and (−) sRNAs detected by qRT-PCR in WT and Xrn2_KD mESCs. C. qPCR analysis of L1_Tf copy-number in WT and Rrp6_KD mESCs. D. L1_ORF2, Tf, Gf and A sub-type mRNAs accumulation detected by qRT-PCR in Xrn2_KD and Rrp6_KD mESCs. E. Accumulation of miR-320 detected by qRT-PCR in WT and Dgcr8_KO mESCs. F. Western analysis of AGO2 accumulation in WT and Ago1,2,3,4_KO_hAgo2 mESCs before and after hAgo2 deletion induced by tamoxifen; CM: Coomassie staining of total protein. G. Accumulation of the Hmga2 and Btg2 mRNAs, respectively targeted by mmu-miR-196a and mmu-let-7a/mmu-miR-132, analyzed by qRT-PCR before and after deletion of hAgo2. H. mRNA accumulation of L1_Tf, _Gf and _A sub-types detected by qRT-PCR before and after hAgo2 deletion. I. mRNA accumulation of a single Tf_L1 subtype located on chromosome 17, analyzed by semi-quantitative RT-PCR before and after hAgo2 deletion. (TIF) [file pgen.1005247.s001.tif]

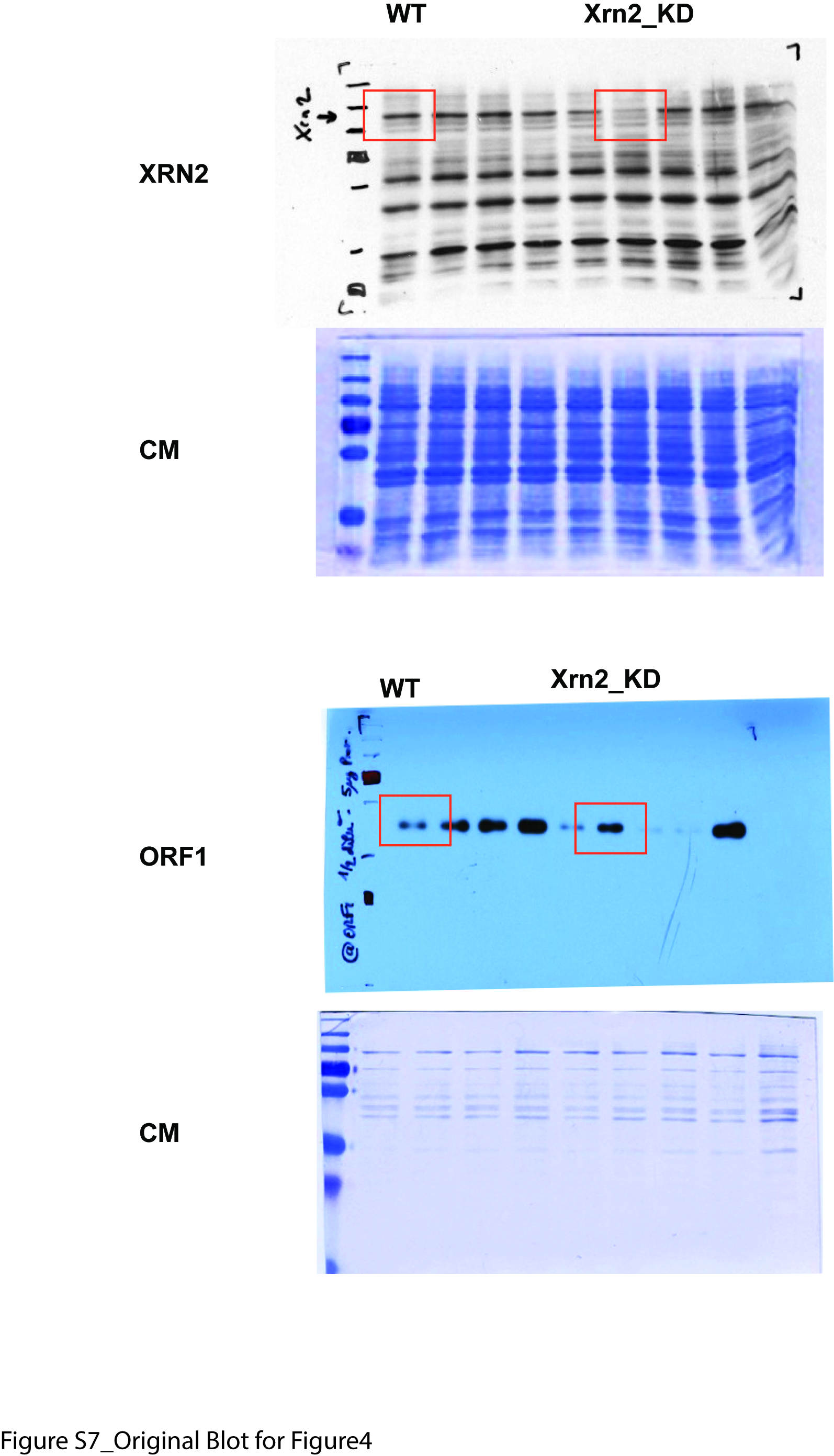

Supplement: S7 Fig — The red rectangles highlight the part of the gel presented in Fig 4A. CM: Coomassie staining of total protein. (TIF) [file pgen.1005247.s002.tif]

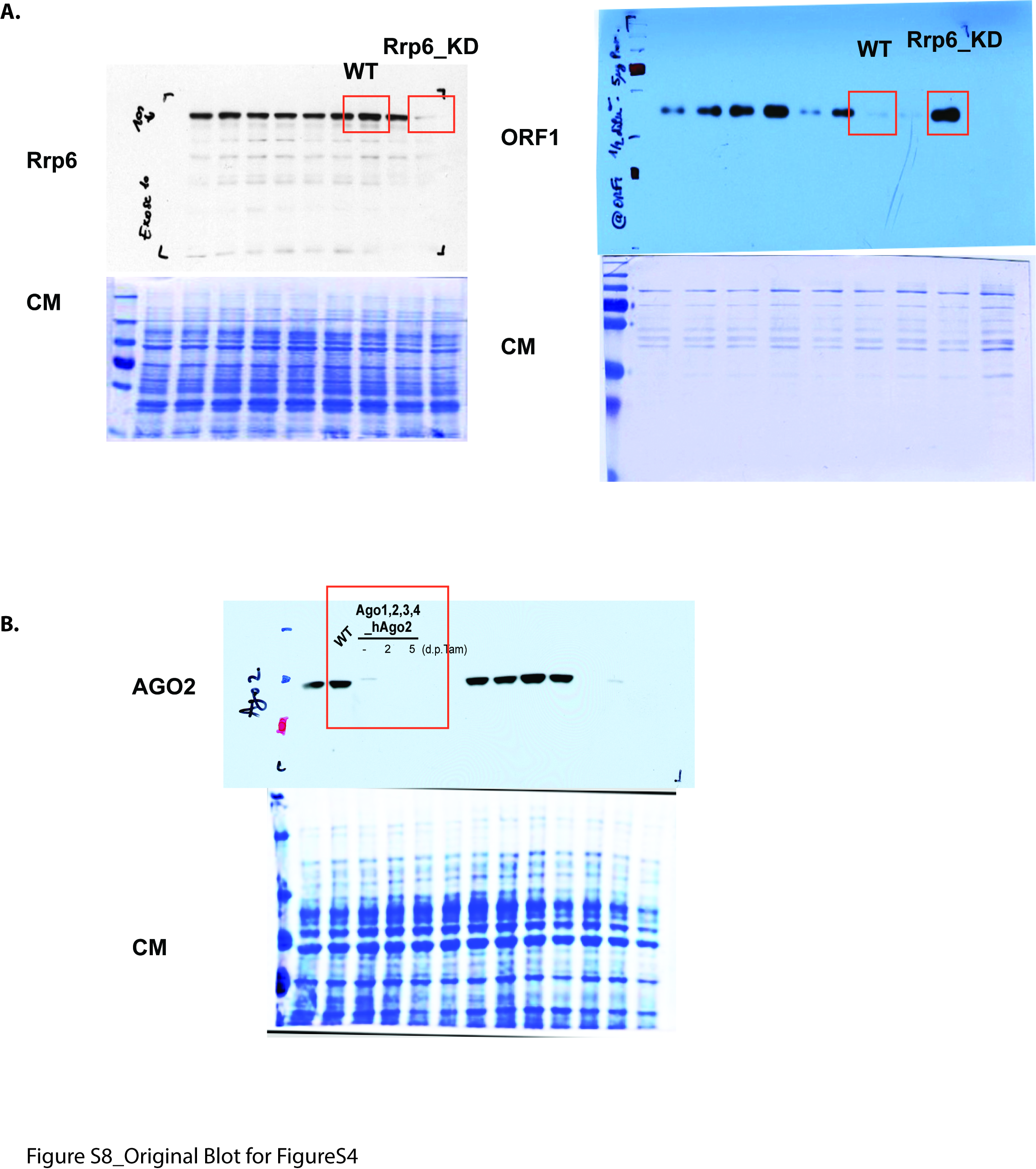

Supplement: S8 Fig — A. Original blot for the S4A Fig. The red rectangles highlight the part of the gel presented in S4A Fig. B. The original blot for the S4F Fig is presented. CM: Coomassie staining of total protein. (TIF) [file pgen.1005247.s003.tif]
